# Recent Coselection in Human Populations Revealed by Protein–Protein Interaction Network

**DOI:** 10.1093/gbe/evu270

**Published:** 2014-12-21

**Authors:** Wei Qian, Hang Zhou, Kun Tang

**Affiliations:** Key Laboratory of Computational Biology, CAS-MPG Partner Institute for Computational Biology, Shanghai Institutes for Biological Sciences, Chinese Academy of Sciences, Shanghai, China

**Keywords:** recent positive selection, PPI network, network topology, coselection, coevolution, pathway selection

## Abstract

Genome-wide scans for signals of natural selection in human populations have identified a large number of candidate loci that underlie local adaptations. This is surprising given the relatively short evolutionary time since the divergence of the human population. One hypothesis that has not been formally examined is whether and how the recent human evolution may have been shaped by coselection in the context of complex molecular interactome. In this study, genome-wide signals of selection were scanned in East Asians, Europeans, and Africans using 1000 Genome data, and subsequently mapped onto the protein–protein interaction (PPI) network. We found that the candidate genes of recent positive selection localized significantly closer to each other on the PPI network than expected, revealing substantial clustering of selected genes. Furthermore, gene pairs of shorter PPI network distances showed higher similarities of their recent evolutionary paths than those further apart. Last, subnetworks enriched with recent coselection signals were identified, which are substantially overrepresented in biological pathways related to signal transduction, neurogenesis, and immune function. These results provide the first genome-wide evidence for association of recent selection signals with the PPI network, shedding light on the potential mechanisms of recent coselection in the human genome.

## Introduction

Approximately 50,000–100,000 years ago, the anatomically modern human migrated from Africa to the rest of the globe. During this period of time, human populations likely underwent a series of local adaptations due to dramatically changing environmental factors such as climate, diet, and pathogens (Sabeti et al. 2006; Nielsen et al. 2007). As a result, populations from different geographical regions have distinct genetic profiles in certain genetic loci, many of which lead to substantial phenotypic variations. Typical examples include the selection sweeps in *SLC24A5* and *MATP* that contribute to population divergence in skin pigmentation (Izagirre et al. 2006; Sturm 2006; Norton et al. 2007) and the nearly fixed difference in *EDAR* that distinguishes East Asians (EAS) from other populations (Sabeti et al. 2007; Fujimoto et al. 2008). One single nucleotide polymorphism (SNP), near the *LCT* gene, is associated with a higher frequency of lactose tolerance in European adults and probably arose during the emergence of dairy farming (Gibson 2007; Tishkoff et al. 2007; Kelley and Swanson 2008; Ingram et al. 2009). Due to the rapid increase in large-scale polymorphism data and the development of new statistical methods, many studies have been done to detect genome-wide signals of positive selection. Several hundred to more than one thousand candidate regions, which may have undergone recent positive selection, have been reported in these studies (Sabeti et al. 2002; Voight et al. 2006; Tang et al. 2007; Akey 2009; Pickrell et al. 2009). The abundant selection signals are in sheer contrast with the relatively short period of time since humans migrated from Africa. If each selection event would produce a single selection signal, it is hard to imagine that there occurred so many independent selection events during the recent human history. Questions have been raised regarding the potentially high false positive rates and the effects of negative selection (Teshima et al. 2006; Hernandez et al. 2011). Nonetheless, a robust method, based on the examination of population differentiation, revealed that a substantial fraction of functional genomic regions have been affected by negative selection and recent positive selection (Barreiro et al. 2008). Furthermore, there is only a short list of local adaptation cases for which molecular biological assays have validated the functional consequences (Grossman et al. 2013; Kamberov et al. 2013). There are even fewer loci that have plausible evolutionary explanations (Akey 2009). This is partially due to the fact that the responses of many candidate genes do not seem to have any direct functional relevance to well-established external selection forces such as changes in climate, diet, or the occurrence of infectious diseases. On the other hand, it is well known that molecular factors do not work in isolation in organisms, but function in a collective manner and are often described as cellular networks (Barabasi and Oltvai 2004; Rual et al. 2005; Han 2008). As reported, recent positive selection in human populations has targeted some biological pathways such as melanin pathway influencing skin pigmentation (Izagirre et al. 2006; Lao et al. 2007; Wilde et al. 2014) and asparagine *N*-Glycosylation metabolic pathway (Dall'Olio et al. 2012). A number of positively selected candidate genes in the same pathways or interaction subnetworks have also been identified. Known examples are *EGLN1* and *EPAS1* in the hypoxia-response pathway playing key roles in genetic adaptation to high-altitude regions (Xu et al. 2011) as well as multiple genes in the NRG-ERBB4 developmental pathway (Pickrell et al. 2009). However, few studies have been done to systematically investigate the relationships between recent selection signals on the whole protein–protein interaction (PPI) network in human populations.

Two different mechanisms may produce such a large number of genes under recent positive selection. The first is coevolution. At intermolecular level, coevolution describes a process where a change in one gene exerts selective pressure on others. It is hypothesized that coevolution may occur via physical interactions, resulting in compensatory changes in the partner proteins in response to a prime functional change in the protein directly under external selection pressure (Juan et al. 2008a; Pazos and Valencia 2008); nonetheless, physical interaction may not be necessary as the effects of functional compensation may be mediated through the enforcement of homeostasis via transcriptional regulation or reaction dynamics/kinetics (Chen and Dokholyan 2006; Kuo et al. 2010). Coevolution of interacting proteins, in large time frames, has been intensively studied and is typically based on the evolutionary distances across different species (Moyle et al. 1994; Pazos and Valencia 2001; Goh and Cohen 2002). Similarities in the evolutionary paths of different genes are often taken as evidence for coevolution and utilized to reconstruct PPI networks (Juan et al. 2008b; Tillier and Charlebois 2009; de Juan et al. 2013). On the other hand, an alternative mechanism notes that epistatic interaction is not compulsory to explain the associated evolutionary patterns. If selection pressures act on an entire pathway or a functional subnetwork, multiple genes in the same pathway/subnetwork may change in the same fitness direction, and at a same evolutionary rate and time to achieve a common phenotypic outcome. Therefore the association in evolutionary patterns may simply reflect parallel selection of different genes in the same pathway of shared functionality (Hakes et al. 2007; Clark et al. 2012). Nonetheless, both hypothetic mechanisms would lead to a set of similar predictions: first, the genes of positive selection would cluster closer to each other in the PPI network than predicted under null hypothesis; second, the clustered genes of selection may share more similar evolutionary paths than genes unrelated on the PPI network. In this study, we do not intend to distinguish between coevolution and parallel selection on the network. Instead, we consider the above two conditions together as coselection and try to examine the evidence for recent coselection in the human genome.

This study of coselection in recent human history is based on the PPI network. Recently, human PPI networks have been constructed via high throughput yeast-two-hybrid (Y2H) experiments and great efforts have been made to improve the accuracy of the interactome data (Rual et al. 2005; Stelzl et al. 2005; Szklarczyk et al. 2011). Many important inferences have been made regarding protein evolution based on PPI networks. Numerous studies reported that proteins located closer to the center of PPI network evolved more slowly than those at the periphery of the network, consistent with the view that central proteins are more essential and receive greater evolutionary constraints (Jeong et al. 2001; Fraser et al. 2002; Yu et al. 2004; Kim et al. 2007). Furthermore, Fraser et al*.* (2002) found that the evolutionary rates of directly interacting proteins tend to be more similar compared to proteins that are far apart. Short-distance force in the yeast protein interaction network has been suggested to drive protein coevolution (Liang et al. 2010). However, all of these studies analyzed network evolution on a macro-evolution timescale (e.g., across different species). At the genetic diversity level within populations, Khurana et al. (2013) found that a model combining the knowledge of biological network and evolutionary properties can well predict the functional essentiality of genes in human diseases. Another study examining the recent positive selection in the insulin/target of rapamycin (TOR) signal transduction pathway showed that selection signals tend to occur on the central genes. Such a pattern is probably specific to this pathway (Luisi et al. 2012). Nonetheless, a comprehensive analysis of the relationship between the network properties and the evolutionary rate in recent human history is necessary to resolve such inconsistencies.

In this study, we examine the dependence of recent positive selection signals on the PPI network regarding 1) network positions and 2) associations between positively selected genes. The genome-wide signals of recent positive selection were scanned in the phase 1 data set of the 1000 Genome Project using a modified composite of multiple signals (CMS) method (Grossman et al. 2010; Abecasis et al. 2012). These selection signals were then mapped onto a human PPI network constructed from the Human Protein Reference Database (HPRD; Keshava Prasad et al. 2009) in which protein interactions were derived manually by a critical reading of the published literature by expert biologists. The network positions of the selected genes were queried using statistics of network centrality; the associations between selection signals were evaluated based on mutual network distance and correlation of divergence trees. Finally, candidate subnetworks enriched with recent coselection signals were identified, and a functional enrichment analysis on subnetwork clusters was performed to make biological interpretation of the clustering of the selection signals.

## Materials and Methods

### Identification of Signals Underlying Recent Positive Selection

Sequencing data of three continents were obtained from the 1000 Genomes Project interim phase 1 data set, including Europeans (CEU; 85 samples), Yorubans (YRI; 88 samples), and EAS (186 samples CHB+JPT). The data is available here: ftp://ftp-trace.ncbi.nih.gov/1000genomes/ftp/release/20101123/interim_phase1_release/, last accessed August 2012.

A modified CMS method was applied to scan for genome-wide signals of recent positive selection, as previously described (Grossman et al. 2010). In brief, lnRsb (based on long haplotype; Tang et al. 2007), ΔDAF (differences in derived allele frequencies), DAF (frequencies of derived alleles), and *F*_ST_ (genetic divergence) were combined to derive a summarized score for each SNP across the genome. The CMS score is defined as follows:

(1) $$\text{CMS}=\prod_{i=1}^{n}\frac{(1-p(s_{1}))x\pi }{\left(1-p(s_{i}))x\pi +p(s_{i})x(1-\pi \right)}$$

where is the empirical *P*-value of the *i*th test. We assume 1% of genome was under positive selection and therefore π=0.01.

A uniform CMS threshold of 10 was used to define the top SNPs of positive selection signals, which corresponded to 0.07–0.16% of total SNPs in the three populations. The top SNPs, if they lie less than 10 kb from each other, were connected to define the candidate regions of positive selection. Regions with only one SNP were removed. The SNP with the highest CMS score in each region was defined as the peak SNP. The gene encompassing the peak SNP was taken as the putative candidate gene of recent positive selection. When no gene overlapped with the peak SNP, the gene closest, and within 20 kb of the peak SNP, was defined as a candidate gene of positive selection (supplementary table S1, Supplementary Material online).

### Human PPI Network

The human PPI data was obtained from the HPRD (Keshava Prasad et al. 2009). Release 9 was used in our study. The file name is HPRD_FLAT_FILES_041310.tar.gz. (http://www.hprd.org/download, last accessed February, 2013). The nodes represent proteins and the physical interactions of the proteins are depicted by edges. The largest connecting network, containing 9,270 nodes and 38,861 edges, was extracted by filtering isolated interactions. Candidate genes of recent positive selection were mapped onto the network. Cytoscape, a network analysis tool, facilitated the visualization of the human PPI network and of integrative information (Shannon et al. 2003).

### Network Position of Recent Positive Selection

Degree centrality (DC) and betweenness centrality (BC) were used to measure both local and global topological positions of candidate genes on the human PPI network (Freeman 1977; Kim et al. 2007). Degree is defined as the number of connections a node has with its neighbors whereas betweenness quantifies the number of times a node acts as a bridge along the shortest path between any other pairwise nodes. The degree and betweenness of node ***v*** can be defined as:

(2.1) DC(v)=N

(2.2) $$\text{BC }\left(v\right)=\underset{s\neq v\neq t}{\sum }\sigma_{\text{st}}(v)$$

Where *N* is the number of first neighbors to the node *v* and $\sigma_{\text{st}}$ is the number of shortest paths from node *s* to *t* that go through *v*.

The Mann–Whitney *U* test (also called Wilcoxon rank sum test) was applied to compare the two centrality measures, BC and DC, between selection signals and nonselection signals, to determine whether network position influences recent positive selection.

The BC and DC values were broken down into seven and six intervals, respectively, keeping a roughly equal number of proteins within each interval, in order to test positive selection signal enrichment in different centrality ranges (fig. 2). The empirical proportion of positively selected genes was compared with 10,000 random resamplings, where proteins of the same number as that of selection candidate list were randomly selected from the network. For each interval of DC and BC, a *Z*-score of the proportion of positive selection was obtained as follows:

(3) $$Z-{\text{score}}_{S,i}=\frac{P_{S,i}-\text{Expected}\left(P_{N,i}\right)}{\text{Standard deviation }\left(P_{N,i}\right)}$$

Where *i* is the interval index, *P_S_*_,_*_i_* is the empirical proportion of selection, and *P_N_*_,_*_i_* is the proportion of randomly sampled proteins under the null hypothesis.

The *P*-values of the intervals were calculated by ranking the empirical proportions against the random sampling. These nominal *P*-values were further corrected for multiple testing using the false discovery rate (FDR; Benjamini and Hochberg 1995). The calculations of DC and BC were carried out using the R package igraph (Csardi and Nepusz 2006).

### Dependence of Signals of Recent Positive Selection on Network Distances

The shortest path length (SPL) was used to represent the interaction distance between any pair of nodes on the PPI network. SPL is the length of the shortest path between a pair of nodes. The average SPL is the average length of the shortest paths for all pairwise nodes on the PPI network.

For each SPL score, the empirical proportion of candidate genes of positive selection was calculated. To bridge the comparisons among different populations, these proportions were further normalized against their corresponding expected proportions assuming no coselection (the number of all coselected interactions divided by the total number of pairwise interactions), Therefore a relative proportion score over 1 indicates an overrepresentation of coselection and vice versa. The Spearman’s correlation test was carried out between the relative proportion scores of coselected genes and the corresponding SPL scores.

The empirical proportions of coselected genes were normalized to obtain *Z*-scores by 10,000 permutations to test the enrichment of coselected genes within each SPL bin. Briefly, in each permutation, a random set of proteins of the same number as the candidate list was selected and the mutual network distances of SPL were calculated. For each SPL score, the empirical proportion of the coselected genes was ranked against those of the permutations to obtain the empirical *P*-value. These *P*-values were further corrected for multiple testing by the FDR method (Benjamini and Hochberg 1995; supplementary table S2, Supplementary Material online).

Similar to equation (3), a *Z*-score of the proportion of genes under positive selection was obtained as follows:

(4) $$Z-{\text{score}}_{S,d}=\frac{P_{S,d}-\text{Expected}\left(P_{N,d}\right)}{\text{Standard deviation}\left(P_{N,d}\right)}$$

Where *d* stands for the individual SPL score, *P_S_*_,_*_d_* is the empirical proportion of coselection, and *P_N_*_,_*_d_* is the proportion of the randomly sampled proteins under the null hypothesis. The calculations of SPL were carried out using the R package igraph (Csardi and Nepusz 2006).

### Tests Based on the Similarities of Divergence Trees

A trifurcate divergence tree was constructed for the three human populations, EAS, CEU, and YRI, for each gene. Briefly, the pairwise molecular *F*_ST_ was calculated for any pair of populations (Weir and Clark 1984; Holsinger and Weir 2009). For each gene, a trifurcate divergence tree was constructed by calculating the branch length of each population from the common ancestor, represented by the divergence rate *D* as follows:

(5) $$D_{i}=\frac{1}{2}(F_{\text{ST}(i,j)}+F_{\text{ST}(i,k)}-F_{\text{ST}(k,j)})$$

where *i*, *j*, and *k* stand for the three populations.

The first test based on divergence trees was the ranked tree distance (RTD). All genes within each population were first ranked by their divergence rate *D*. The absolute rank distance between any two genes, *a* and *b*, in the population *i* is therefore $\left|\text{Rank}\left(D_{\mathrm{ai}}\right)-\text{Rank}(D_{\mathrm{bi}})\right|$. RTD is simply the arithmetic average of the distances in the three populations:

(6) $$\text{RTD}=\frac{1}{3}\overset{3}{\underset{i=1}{\sum }}\left|\text{Rank}\left(D_{\mathrm{ai}}\right)-\text{Rank}(D_{\mathrm{bi}})\right|$$

RTD was further normalized to have a zero mean and unit variance.

The second test was the synergic score (Syn score). For a single gene, the signed difference of its divergence rate *D* from the genome median *D****^′^*** in one population roughly indicates the strength and direction of the underlying evolution. For a pair of genes, *a* and *b*, the Syn score is the weighted average product of this residual divergence rate (*D-D*^′^) as follows:

(7) $$\text{Syn}=\frac{1}{3}\overset{3}{\underset{i=1}{\sum }}\frac{\left(D_{ai}-D^{\prime }{}_{i})\times (D_{bi}-D^{\prime }{}_{i}\right)}{{\text{SD}}_{i}{}^{2}}$$

where *i* is the population and SD*_i_* is the standard deviation of divergence rate in the population *i*.

The statistical significance of the empirical distributions of RTD and Syn were evaluated by comparison with 1,000 sets of permutations, in which the divergence rates *D_i_* were randomly reshuffled within each population among all the PPI nodes.

### Functional Enrichment Analysis

The DAVID Bioinformatics Database (http://david.abcc.ncifcrf.gov/, last accessed July, 2013) was used to perform functional enrichment analysis on the clusters of coselection subnetworks. Databases assayed for enrichment include gene ontology, PANTHER, KEGG pathway, and SP-PIR keywords of functional category and tissue expression (Huang da et al. 2009a,b). The Benjamini FDR method was used to correct for multiple testing (Benjamini and Hochberg 1995).

## Results

### Signals of Recent Natural Selection

**Fig. 1.—:**
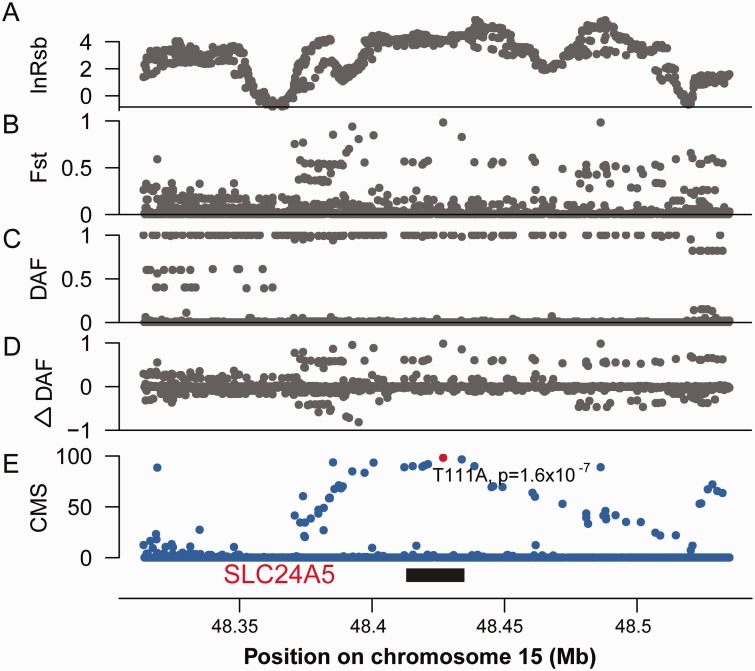
Selection signals of *SLC24A5* in CEU detected by CMS. Four different statistics (*A*) lnRsb (*B*) *F*_ST_ (*C*) Derived allele frequency and (*D*) ΔDAF were calculated and plotted for the 100 kb region centered around *SLC24A5*. (*E*) CMS score integrated all four signals above into a single value. A higher CMS score indicates a stronger selection signal. The red dot denotes the CMS score for the nonsynonymous SNP, *rs1426654*, which is generally accepted as a major causal variant responsible for the fairer skin pigmentation in CEU. It can be seen that *rs1426654* has the highest CMS score within this region and corresponds to a highly significant *P*-value.

We carried out a genome-wide analysis of signals of positive selection in three groups of humans: The YRI from Africa, CEU and a combined panel of EAS composed of Chinese and Japanese (EAS). The 1000 Genomes Project phase 1 data (Abecasis et al. 2012) were used. We applied a CMS method to scan for genome-wide signals of recent positive selection. The CMS is considered to be state-of-art and provides a better estimation of the centers of selection (Grossman et al. 2010). In this study, the original CMS statistic was modified to combine the signals of lnRsb, ΔDAF, DAF, and *F*_ST_ in order to obtain a summarized score for each SNP. The adjacent SNPs of the top CMS scores were combined to identify the candidate regions of positive selection (fig. 1), and the peaks of regions were mapped to genes that represented the most likely target of selection (see Materials and Methods). Figure 1 shows the selection signals of *SLC24A5* in each single test and in the combined CMS test. *SLC24A5* is a well-established example of recent local adaptation and is a major genetic factor that contributes to the light skin pigmentation in CEU (Lamason et al. 2005; Izagirre et al. 2006). In total, 2,462, 1,604, and 1,411 of the candidate genes potentially affected by positive selection were identified, and 983, 663, and 591 of them were successfully mapped onto the human PPI network in CEU, EAS, and YRI, respectively (supplementary table S1, Supplementary Material online). It should be noted that in this study, we considered much more candidate regions than previous scans of recent selection. Our method is mainly based on ranking the CMS scores, thus we used a relatively relaxed cutoff (CMS = 10) to define the top SNPs. The lower cutoff will surely increase the false positive rate, but will increase the chance of detecting weaker positive signals as well. We chose such a tradeoff because our main purpose is to detect the global association among the selection signals. The inclusion of more false positives, which arise from the null distribution, would not change the underlying correlations; however, if too many true positive signals were missing, it would lead to substantial reduction of the test power.

We used the population divergence level, estimated by *F*_ST_, as a stand-alone signal of natural selection. Population divergence reflects how fast subpopulations differentiate from a common ancestral population because different selection forces shape the divergence patterns differently. Local adaptation accelerates the divergence of a specific subpopulation from the other. Balancing selection increases the overall divergence rates, whereas constrained divergence likely indicates purifying selection (Barreiro et al. 2008). Molecular *F*_ST_ was calculated for every gene (see Materials and Methods).

### Network Position of Recent Positive Selection

First, we studied whether or not signals of recent positive selection depend on the PPI network. The DC determines the number of neighbors that directly interact with a protein and is a measurement of local centrality. BC measures the likelihood a protein intercepts the shortest paths of any random pairs of nodes (see Materials and Methods). It is a measurement of global centrality. DC and BC were calculated for all proteins in the PPI network, and the nonparametric Mann–Whitney *U* test was used to examine whether the centrality scores were different between the selection and nonselection status (1 = a gene with selection signal and 0 = no selection signal). In general, there was a weak difference in the centrality scores between the genes of selection and nonselection. Such a trend was most obvious in EAS (DC: *P* = 0.002; BC: *P* = 0.007) or when all three populations were combined together (DC: *P* = 0.005; BC: *P* = 0.051). YRI had weaker signals (DC: *P* = 0.019; BC: *P* = 0.183) and there was no clear evidence of correlation in CEU (DC: *P* = 0.768; BC: *P* = 0.742).

**Fig. 2.—:**
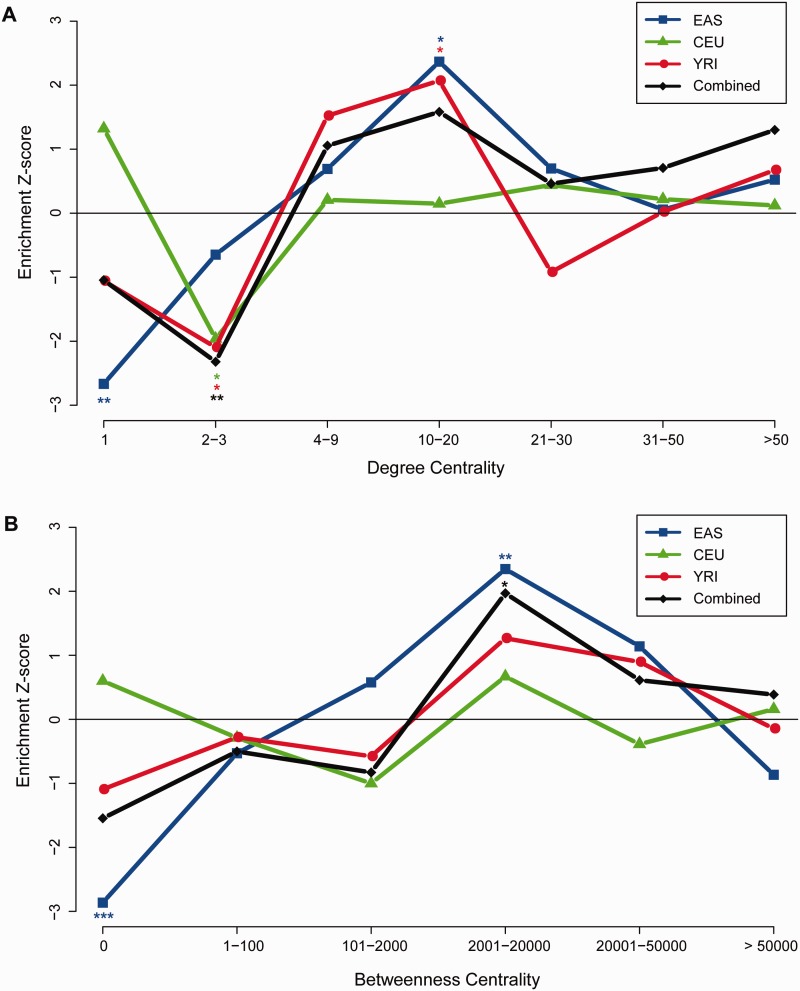
Distribution of signals of recent positive selection on different positions of the human PPI network. (*A*) Distribution conditioned on DC. (*B*) Distribution conditioned on BC. In both plots, the *x* axes represent different intervals of centralities, either as DC scores in (*A*) or as BC scores in (*B*). The *y* axes represent the *Z*-scores of proportions of recent positive selection against null distribution of 10,000 random samplings. Lines in different color represent different populations. EAS, Han Chinese and Japanese; CEU, Europeans; YRI, Yorubans; combined, combination of the three populations. * indicates *P*-values < 0.05, ** < 0.01, and *** < 0.001.

To look into more details, we calculated the proportions of selection genes within different intervals of centrality scores. The observed proportions of selection genes were normalized by 10,000 random permutations to derive a *Z*-score for each interval, and then they were ranked against the null distribution generated by permutation for statistical significance (see Materials and Methods). As seen in figure 2*A*, signals of selection tended to be underrepresented for genes with fewer neighbors. Genes with DC 1 in EAS were strongly underrepresented (nominal *P* = 0.003) and genes with DC scores of 2 and 3 were underrepresented in CEU (nominal *P* = 0.025) and YRI (nominal *P* = 0.015). There seemed to be a moderate enrichment of selection signals in genes with an intermediate or high number of network neighbors (DC interval of 10–20) in EAS (nominal *P* = 0.012) and YRI (nominal *P* = 0.022), but this enrichment was not clear in CEU (nominal *P* = 0.437). The proportion of selection genes was not obviously enriched compared with the permutation in genes with very high degrees (degree > 20, fig. 2*A*), likely due to the limited number of high degree genes (only 8.8% of genes had a degree > 20). The degree analysis for all three populations combined showed a similar trend: the signals of selection were underrepresented in the lower range (DC 2–3, nominal *P* = 0.01), but were mildly enriched in the intermediate to high range (DC 10–20, nominal *P* = 0.054). It should be noted that, after multiple testing corrections, only the DC of 1 and DC of 10–20 in EAS was still significantly different from the null distribution (FDR corrected *P* = 0.023 and 0.043 respectively, supplementary table S2, Supplementary Material online; see Materials and Methods).

A similar pattern was observed when the proportions of selection were conditioned on the BC score of global centrality (fig. 2*B*). In EAS, the proportions of selection increased from a significant underrepresentation at the very periphery of the network (BC = 0; nominal *P* = 0.001, FDR corrected *P* = 0.007) to a marginally significant overrepresentation with high BC scores of 2,001–20,000 (nominal *P* = 0.008, FDR corrected *P* = 0.025). YRI and the combined data of three populations showed a similar but insignificant trend (fig. 2*B*). The combined populations showed an enrichment of selection signals at a relatively high BC range (BC of 2,001–20,000, nominal *P* = 0.024, not significant after multiple testing correction). A clear trend was lacking for the CEU population in the BC analysis (fig. 2*B*).

In brief, there was a weak trend of network position dependency in the recently selected genes. Selection signals tend to enrich at the subcentral positions of the PPI network, particularly in EAS. However, such a trend was not well supported by formal statistical tests, suggesting that the dependence of recent positive selection on PPI network is weak at most if not being totally false. On the other hand, the likely enrichment of recent positive selections at the subcentral positions can be explained: Genes with protein products of very high centrality could be under strong evolutionary constraints and hence accommodate fewer selective events, whereas genes at the periphery may not contribute strongly enough to phenotypic effects for selection due to their network locations. Given above, it seems that subcentral proteins may have achieved an optimal tradeoff between functional constraints and adaptive effects, and thus were more targeted by adaptive selection in the recent human evolution.

### Genes under Recent Positive Selection Lie Closer to Each Other on the PPI Network

Either coevolution or pathway selection predicts that coselected genes should lie closer to each other in the interactome than expected when there is no coselection. To test this hypothesis, we studied the relationship between the distribution of signals of positive selection and their distances on PPI network. The average SPL was used as the measurement of network distance between pairs of proteins. Empirical *P*-values were obtained by comparing the average SPL score between a pair of candidate genes of selection to those between a pair in random samples (10,000 times) (see Materials and Methods). We found that the average SPL value between a pair of candidate genes, both carrying signals of recent positive selection (hereafter referred to as coselection), was 4.06 in EAS and 4.09 in YRI; both significantly shorter than the expectation of 4.23 under null hypothesis (*P* < 10^−^^4^ in EAS and *P* = 0.002 in YRI; supplementary figs. S1 and S3, Supplementary Material online). Coselection in CEU also showed a smaller average SPL of 4.18, but this was not statistically significant (*P* = 0.066, supplementary fig. S2, Supplementary Material online). When the three populations were evaluated together, genes that have signals of positive selection were found to lie closer to each other than expected under null distribution (mean SPL value of 4.14; *P* < 10^−^^4^; supplementary fig. S4, Supplementary Material online). This provided clear evidence indicating closer interactions among genes underlying recent positive selection than expected.

**Fig. 3.—:**
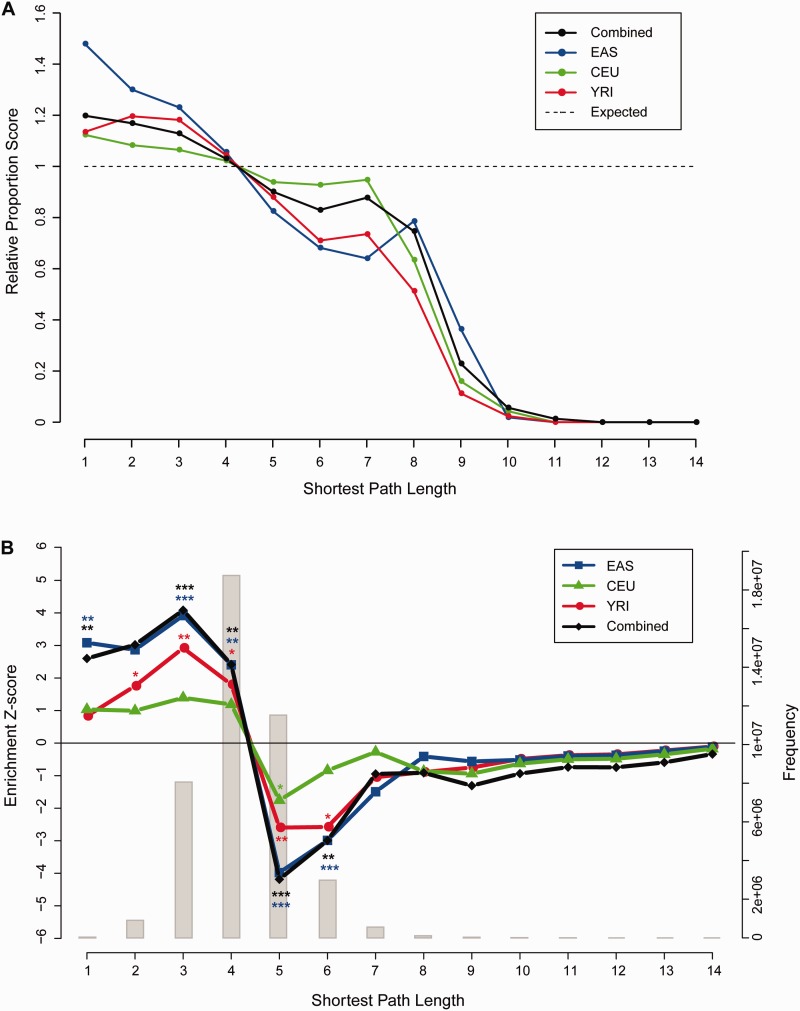
Dependence of signals of recent positive selection on PPI network distances. (*A*) Proportions of coselected genes over network distance of SPL in CEU, YRI, EAS, and three populations combined and normalized against the expected proportions assuming no coselection (dotted line). (*B*) The *Z*-score of coselection (left *y* axis) is plotted against SPL distances. The frequencies of pairwise interactions of the whole PPI network are plotted against SPL as a background histogram in light gray color. Lines in different colors represent different populations as described in figure 2. * stands for *P*-values < 0.05, ** < 0.01, and *** < 0.001.

To further examine this pattern, we scrutinized the proportion of coselection interactions for every SPL distance. The relative proportion score of coselected genes decreased almost monotonically as the network distance increased (fig. 3*A*). The negative correlations between the coselection scores and SPL were highly significant in all four groups (*P* = 7.8 x 10 ^−^^9^, 2.9 x 10^−^^9^, 2.9 x 10^−^^9^, and 6.7 x 10^−^^12^ for YRI, EAS, CEU, and combined, respectively). Furthermore, the negative relationship could also be clearly observed when the *Z*-scores that were obtained from permutation (see Materials and Methods) showed enrichment of coselection over network distances. Specifically, pairs of positively selected genes tended to interact more often over SPL of 1–4, than expected under the null hypothesis (fig. 3*B*), especially in EAS (nominal *P* = 0.003, 0.004, 10^−^^4^, and 0.01, FDR corrected *P* = 0.011, 0.012, 7 x 10^−^^4^, and 0.023 for SPLs of 1–4, respectively) and in the combined populations (nominal *P* = 0.007, 0.002, 10^−^^4^, and 0.007, FDR corrected *P* = 0.017, 0.007, 7 x 10^−^^4^, and 0.017 for SPLs of 1–4, respectively). Such a trend was moderate in YRI and weaker in CEU (fig. 3*B* and supplementary table S2, Supplementary Material online). Reciprocally, the proportions of coselection interactions quickly decreased below the neutral level at SPL of 5 (nominal *P* = 0.006, 10^−^^4^, 0.043, and 10^−^^4^, FDR corrected *P* = 0.026, 7 x 10^−^^4^, 0.321, and 7 x 10^−^^4^ for YRI, EAS, CEU, and combined, respectively) and at SPL of 6 (nominal *P* = 0.004, 8 x 10^−^^4^, 0.203 and 0.001, FDR corrected *P* = 0.026, 0.004, 0.321 and 0.006 for YRI, EAS, CEU and combined, respectively, fig. 3*B*).

The shorter SPL distances between genes underlying positive selection seem to be consistent with the effects of coselection. However, other factors may also confound the SPL distribution. First, positively selected genes might have occurred more often toward the subcenter of the PPI network (fig. 2). Therefore, the correlation between coselection and SPL may result from the varying likelihood of selection at different network positions. To control for this effect in the current study, the PPI network was divided into three separate parts: the peripheral subnetwork (BC ≤100), the intermediate (100 < BC ≤ 20,000), and the core (BC >20,000). The SPL analysis for coselection was restricted within each subnetwork, where the SPL distances between each pairs were the same as in the global PPI network. The negative correlation between the SPL and relative proportion score of coselection well persisted in the peripheral subnetwork across all four groups (Spearman’s rho = −0.93, −0.96, −0.98, and −0.95, *P* = 3.4 x 10^−^^6^, 1.2 x 10^−^^7^, 1.6 x 10^−^^8^, and 5 x 10^−^^7^ for EAS, CEU, YRI, and combined respectively, supplementary fig. S7*A*, Supplementary Material online). *Z*-scores of coselection also mimicked the pattern in the full data set (fig. 3*B*) and showed statistical significance (supplementary fig. S7*B*, Supplementary Material online). As for the intermediate subnetwork, we observed a clear negative correlation between relative proportion scores of coselected gene pairs and SPL distances in EAS, YRI, and combined populations (supplementary fig. S6*A*, Supplementary Material online, *P* < 0.05, supplementary table S3, Supplementary Material online). They also showed significant overrepresentation over smaller SPL distances of 1–3 and underrepresentation over those larger than 4 (supplementary fig. S6*B*, Supplementary Material online). In the core subnetwork, however, there was no strong evidence of negative correlation between the coselection scores and SPL, although marginal signal of negative correlation was found in EAS (supplementary fig. S5*A*, Supplementary Material online, *P* = 0.02, supplementary table S3, Supplementary Material online). Figure S5*B* showed a similar general trend of coselection enrichment to that in the full data set (fig. 3*B*), but such trend was not well supported by the statistical *P*-values (supplementary fig. S5*B*, Supplementary Material online). This suggests that there may lack cases of coselection around the network core. On the other hand, this may be due to the limited test power given the small number of genes in the core subnetwork.

Second potential confounding factor is the genomic linkage disequilibrium (LD) between the genes of positive selection signals. Functionally related genes are often localized close to each other in the genome and gene families often cluster together. On the other hand, the genomic regions carrying signals of recent positive selection usually span substantial physical distances, up to tens or hundreds of kilo base pairs, and cover multiple genes (Tang et al. 2007). It is therefore possible to assign a single selection signal to multiple functionally related genes. But in our study, most candidate regions (87%) corresponded to single genes. The effects of genomic LD were further filtered out by eliminating all of the one-step PPI interactions where the two partner genes were localized within 500 kb from each other. This did not change the negative correlation between relative proportion scores of coselection and SPL distance (*P* < 1.0 x 10^−^^7^, supplementary fig. S8 and table S3, Supplementary Material online).

Another possibly confounding factor is the gene length. We noticed that there was a weak but significant positive correlation between gene length and the CMS scores in CEU and EAS (CEU: Spearman’s rho = 0.11, *P* = 1.5 x 10^−^^6^; EAS: Spearman’s rho = 0.09, *P* = 0.002), but not in YRI (Spearman’s rho = 0.03, *P* = 0.29). This seems to be mainly caused by a few extremely long genes which happen to overlap with the peak CMS signals (supplementary fig. S9*A*, Supplementary Material online). We then tried to eliminate the effects of gene length in CEU and EAS by changing the way of defining the candidate genes of selection. First, all the genes were surrogated by their central positions in the genome, and therefore the gene length information was removed. Second, each candidate region that carries strong CMS signals was also deduced to a single genomic position where the highest CMS score occurs. A single gene was then assigned to each candidate region if its center is closest to the highest CMS position, given that the distance between the two positions was below a threshold. The distance threshold was fixed to be 150 kb, which allowed the total numbers of candidate selection genes mapped on the PPI network (CEU: 970 genes, CHB: 657 genes) to approximate those found in the main analyses. This scheme did remove the correlations between the gene length and the CMS scores (CEU: Spearman’s rho = 0.036, *P* = 0.106; EAS: Spearman’s rho = 0.014, *P* = 0.618) and it can be seen that most long genes were removed from the analysis (supplementary fig. S9*B*, Supplementary Material online). Nonetheless, we observed highly consistent patterns that the coselected genes remained to cluster strongly in shorter network distances (supplementary fig. S9*C* and *D*, Supplementary Material online). The negative correlations between coselection and SPL remained strong and highly significant for this candidate list (supplementary fig. S9*C*, Supplementary Material online, CEU: Spearman’s rho = −0.96, *P* = 3.8 x 10^−^^8^; EAS: Spearman’s rho = −0.98, *P* = 1.6 x 10^−^^9^). Similarly, coselection genes persisted to interact more often over SPL of 1–4, than expected under null distribution, given by 10,000 permutations (supplementary fig. S9*D*, Supplementary Material online). Especially in EAS, such trend was highly clear (nominal *P* = 0.027, 0.018, 5 x 10^−^^4^ and 0.014, FDR corrected *P* = 0.063, 0.051, 0.004 and 0.048 for SPL of 1–4 respectively), similar to the pattern in the main figure 3*B*. These analyses demonstrated that the gene length may have some confounding effects on the patterns of coselection, but such an effect is marginal at most, and would not determine the overall patterns of coselection.

### Similarity of Divergence Trees

**Fig. 4.—:**
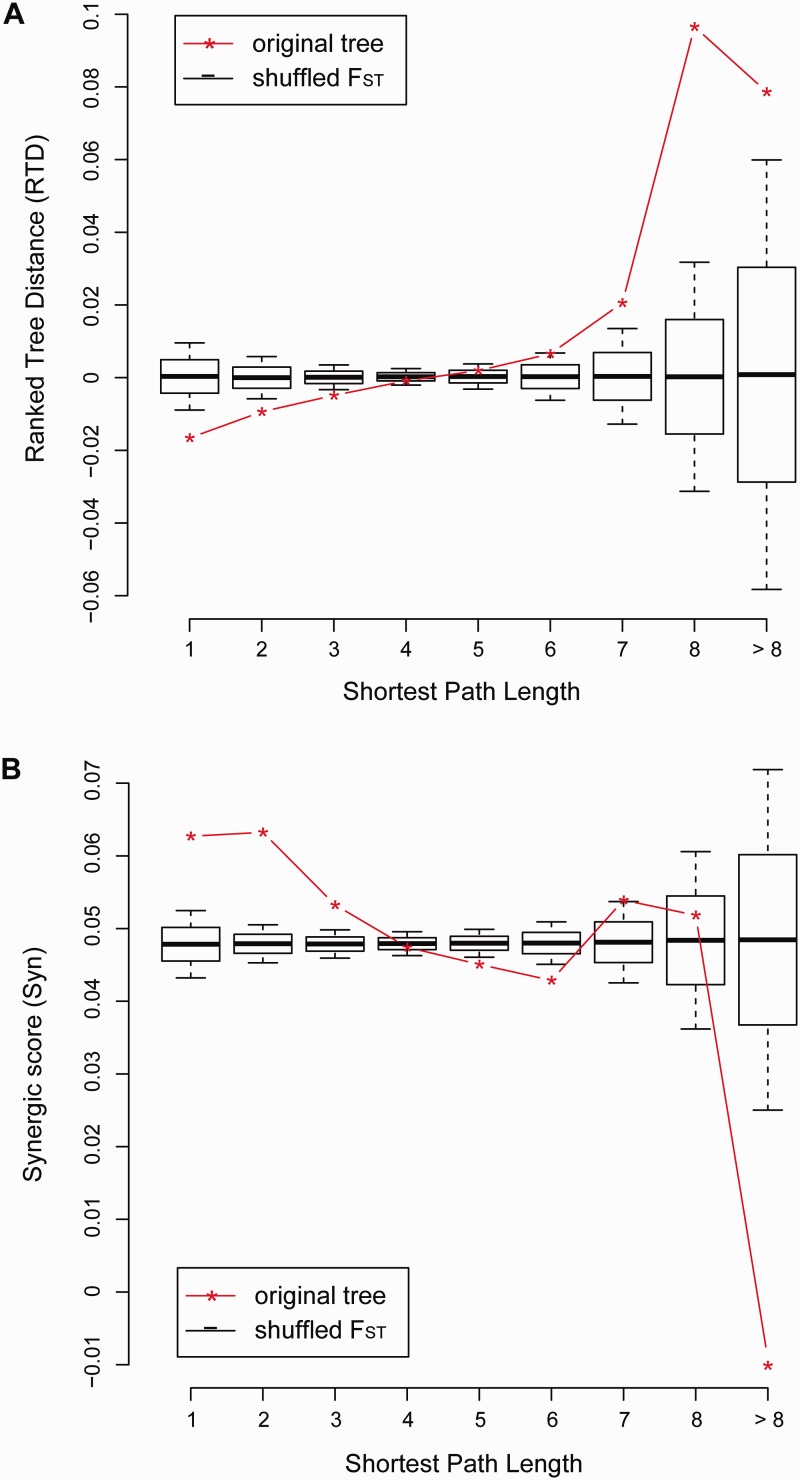
The dependence on PPI network distance of similarities in trifurcate divergence trees between two genes. The similarity of trifurcate divergence trees was calculated either as (*A*) the ranked tree differences (RTD) or (*B*) the Syn score for pairs of genes. The average RTD or Syn scores was plotted against the SPL values, shown as star-linked red lines, and compared with the null distributions generated by random reshuffling as shown by box-plots where the solid black lines represent the expected value of RTD or Syn score for pairwise genes.

The above analysis mainly focused on genes that revealed substantial signals of recent local adaptation as identified by CMS. However genes undergoing weak or more ancient positive selection may not carry the typical patterns of positive selection identifiable by CMS. In previous studies, similarities of evolutionary histories between two molecules were estimated based on evolutionary distances across different species or namely phylogenetic trees. The excessive similarity in evolutionary histories provided abundant evidence for correlated evolution in the large timescale (Pazos and Valencia 2001; Juan et al. 2008a). In the micro-evolutionary timescale, the evolutionary histories can be represented by divergence trees instead. As there are three populations in our study, a trifurcate tree of divergence can be computed based on the pairwise *F*_ST_ distances for each gene (see Materials and Methods), where the edge length of each branch estimates the divergence rate from the ancestral population. Under the null hypothesis of unrelated evolution between genes, there should be a base level of tree similarity between any two genes, due to the shared demographic history. However the pairwise tree similarity should be independent of the interaction network (fig. 4). On the other hand, coevolving genes or genes under pathway selection should have correlated evolutionary paths in a synergic way. As coselected genes also tend to reside close to each other, one expects to see averagely higher tree similarities between genes closer in interactome distance compared with genes that are farther apart. To test this, we therefore constructed two tests based on divergence tree. In the first test, all of the genes were ranked by their divergence rates within each population. The absolute difference in ranks between two genes reflected the similarities in their evolutionary histories, as previously described (Fraser et al. 2002). The rank differences among the three populations were averaged to derive the RTD (see Materials and Methods). Obviously RTD decreases when the tree similarity increases and vice versa. RTD was calculated for all pairs of genes and plotted against the SPL distance. We discovered that RTD was clearly positively correlated with SPL (Spearman’s rho = 0.98, *P *= 5 x 10^−^^5^, fig. 4*A*), suggesting that genes involved in closer mutual protein interactions tend to have evolutionary histories that are more similar.

In order to consider full information of the divergence tree, we conducted the other test, called Syn score (see Materials and Methods). The Syn score measures how much two divergence trees deviate in the same direction from the genome median tree. Specifically, in each population, when a pair of genes both diverged a lot more or a lot less than the genome-wide median divergence tree, the Syn score increased. If the divergence patterns of a pair of genes deviated in opposite directions from the genome median, the Syn score decreased (see Materials and Methods). The average Syn scores were calculated for all pairs of genes and were compared with the null distribution. The null distribution was generated by randomly shuffling branches among the divergence trees within each population (see Materials and Methods). As shown in figure 4*B*, in general, the average Syn score decreased with increasing SPL distance, except that Syn scores associated with SPLs of 7 and 8 were higher than those associated with the SPL of 6. There was a weak negative association between Syn score and SPL (Spearman’s rho = −0.67, *P* = 0.06). Within bins of SPL, Syn scores exhibited some substantial deviations from the null distribution. For small SPLs of 1 and 2, the average Syn scores were significantly higher than the null distribution level (*P ≤ *10^−^^3^), indicating that closely interacting genes did positively influence each other’s evolutionary histories. This trend seemed to be opposite at SPLs of 5, 6, and greater than 8, where the average Syn scores were lower than the null distribution level (fig. 4*B*).

The dependence of tree similarities on network distances of SPL was examined for possible confounding effects due to the genome LD. Briefly, pairwise interactions, in which genes were within 500 kb of each other, were removed from the PPI network. The RTD scores remained strongly positively correlated with the SPL network distance (Spearman’s rho = 1, *P* = 4 x 10^−^^4^, supplementary fig. S10, Supplementary Material online). However, the negative correlation between Syn scores and SPL distance was weaker (Spearman’s rho = −0.68, *P* = 0.11), and the deviation from the null distribution in each SPL bin was insignificant (supplementary fig. S11 and table S3, Supplementary Material online). This suggested that the similar divergence patterns could be partially explained by their mutual influences via physical LD. It should be noted that the physical proximity on the genome does not rule out the possibility of two genes being coselected. In fact, a closer physical distance may serve to amplify the effects of selection (Pavlidis et al. 2012).

### Subnetworks Showing Evidences of Clustering of Recent Coselection

**Fig. 5.—:**
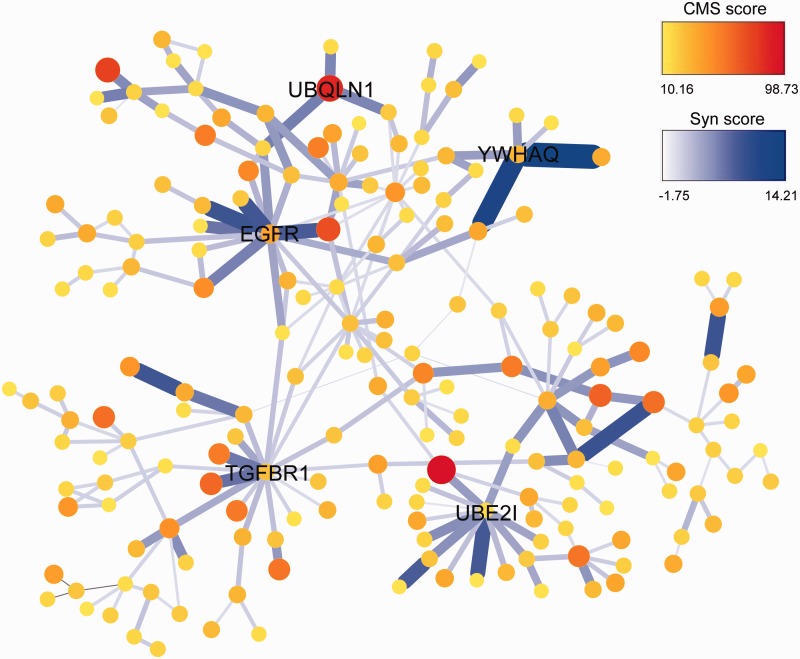
The biggest subnetwork of coselection constituted by signals of recent positive selection in East Asians. The color and size of each vertex is proportional to the corresponding CMS score, whereas the color and thickness of each edge is proportional to Syn score of the interacting pair of proteins. Obvious hub genes are labeled with gene symbols.

Globally, we found strong evidence for coselection in recent human history. Therefore, an investigation was done to determine clusters of genes that have been coselected. The selection genes that closely interacted on the PPI network and shared similar evolutionary histories were considered to be good candidates. First, we extracted the subnetworks that were composed of candidate genes of recent positive selection. Intriguingly, the candidate genes constituted large subnetworks via single-step interactions in all three populations (fig. 5 and supplementary figs. S12 and S13, Supplementary Material online). In coselection clusters, we put forward two scenarios that could be equally possible. First, it is likely that a single protein was directly under external selection pressure (hereby defined as the leading protein). The leading protein would likely undergo the strongest genetic changes given its direct response to the environmental pressures. These changes would then exert internal selection pressures on the interacting partners, likely via functional compensation (Lovell and Robertson 2010), leading to adaptive phenotypic effect based on propagation of genetic changes. Second, the whole biological pathway or functionality module was directly under external selection pressures and proteins involved were all affected, to more or less extent. The resulting local adaptation of phenotypic changes may be contributed by parallel changes in multiple genes involved in this pathway.

**Fig. 6.—:**
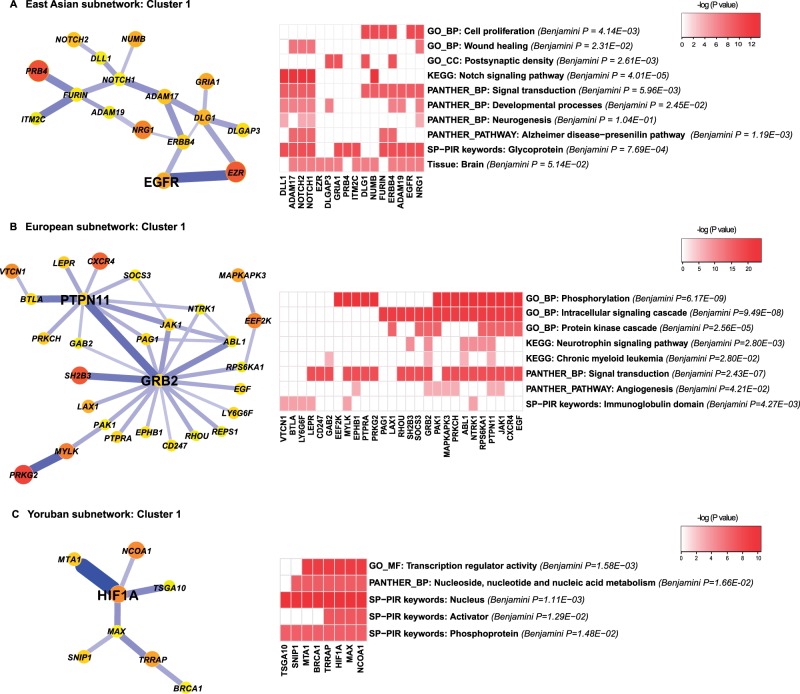
Candidate pathways of recent coselection identified by functional enrichment analysis in (*A*) EAS (*B*) CEU, and (*C*) YRI. In each subfigure, the coselection cluster is plotted at the left. The right side shows a 2-D heatmap of the functional enrichment matrix. For each functional category in a row, the nominal *P*-values of enrichment are log transformed and are shown as a color gradient. Benjamini corrected *P*-values are annotated right to the functional categories. Genes from the clusters, in the columns of the heatmap are either labeled with the color gradients if they appear in the corresponding functional category or left unlabeled if not.

The biggest subnetwork of positively selected genes was plotted in each population. The node size and color gradient were proportional to the CMS score to indicate selection intensity and the thickness and color gradient of the edges were proportional to the Syn score to reflect the relatedness of evolutionary histories between two genes that encode interacting proteins (fig. 5 and supplementary figs. S12 and S13, Supplementary Material online). For example, the EAS subnetwork formed by the candidate genes under positive selection showed some interesting patterns. First, relatively strong interactions (high Syn scores) were usually clustered around some hub genes, such as *TGFBR1*, *UBE2I*, epidermal growth factor receptor (*EGFR*), *UBQLN1*, and *YWHAQ*. Second, the strongest selection signals (high CMS scores) seemed to also reside on these clusters. Third, the strongest signals of selection rarely occurred on the hub (fig. 5). In the *TGFBR1* cluster, *STK35* had the highest CMS signal; *EZR* was the strongest signal in the *EGFR* cluster; *RANBP2* showed the strongest signal in the *UBE2I* cluster; and *LYST* had a higher CMS score than the others in the *YWHAQ* cluster (supplementary figs. S14–S16, Supplementary Material online). Such patterns support the first scenario in which one protein, under strong external selection, may have affected the genetic profiles of a group of proteins via a nearby hub protein, despite that *UBQLN1* had the highest CMS score in its own cluster. The other scenario was also observed that, for instance, the four genes *PIK3CA*, *APPL1*, *RABSA*, and *EEA1* formed a special sequential cluster and had selection signals that were almost equal in magnitude (supplementary fig. S16, Supplementary Material online). The cluster in figure 6*A* was composed of many sequential gene products in highly weighted bonds as well as in similar strength of selection signal, consistent with selection signals in NRG-ERBB4 developmental pathway (Pickrell et al. 2009). Similar patterns were observed in YRI and CEU, although the size of the subnetwork was smaller in YRI and larger in CEU. In CEU, clusters of strong interactions indicating coselection emerged around protein tyrosine phosphatase nonreceptor type 11 (*PTPN11*), growth factor receptor-bound protein (*GRB2*), Cadherin-associated protein, beta 1 (*CTNNB1*), *YWHAG*, *SPTAN1*, and *GNAI2* as well as a sequential cluster of coselection including *LUC7L2*, *MAN2A2*, *NSF*, *C14orf1*, and *ALDH2* (supplementary figs. S12 and S17, Supplementary Material online). In YRI, only genes around hypoxia inducible factor 1 (*HIF1A)* had strong interactions of high Syn scores, but other several interacting pairs of moderate coselection still existed (supplementary fig. S13, Supplementary Material online).

The fact that in YRI we observed fewer clusters of coselection may be partially due to the fewer candidate genes identified in YRI. However, we identified similar numbers of candidate genes in EAS (663) and YRI (591). Interestingly, the number of coselection clusters in EAS is still substantially higher than in YRI. This suggests that there is a difference in the properties of coselection between EAS and YRI, more specifically that selection signals in EAS might extend much shorter network distances than those in YRI, as suggested in figure 3.

### Functional Enrichment of Coselection Clusters in Human Populations

Coselection may result in enriched selection signals in certain biological pathways. Because strong interactions that illustrated similar evolutionary path tend to form clusters, as shown in the previous section, it would be interesting to examine the functional enrichment in these clusters. We extracted subnetworks, connected by one-step edges whose Syn scores are higher than 1.5 for EAS and CEU (accounting for 35% and 23% of coselection interactions, respectively). A slightly lower threshold of 0.76 for Syn score (13% of coselection interactions) was used for YRI, because there were fewer strong interactions. As expected, numerous clusters were naturally formed by the stronger one-step interactions, often centered on hub genes of higher degrees (fig. 6 and supplementary figs. S14–S20, Supplementary Material online). We applied DAVID functional annotation to the largest coselection clusters identified in each population (see Materials and Methods).

In East Asian population, a group of positive selection genes, as an example of coselection, conveyed both scenarios of compensatory changes through hub protein interactions and of being parallel targeted in one pathway. They clustered around *NOTCH1* (notch homolog 1) and *DLG1* (disks large homolog 1) and overlapped with the *EGFR* pathway. Functional enrichment suggested strong involvement of this cluster in the Notch signaling pathway (Benjamini corrected *P* = 4.01 × 10^−^^5^), which plays a major role in neurogenesis and neuronal differentiation (fig. 6*A*). The involvement of this cluster in neural development was further supported by its significant enrichment in postsynaptic density, the Alzheimer’s disease-presenilin pathway and the brain (Benjamini corrected *P* = 0.003, 0.001, and 0.05 respectively, fig. 6*A*). This suggests that the recent coselection in EAS might have specifically targeted the nervous system. Interestingly, this cluster was mostly composed of glycoproteins (12 out of 16 genes, Benjamini corrected *P* = 0.0008) and a group of membrane proteins, which mainly contribute to intercellular signaling. The second cluster in EAS grouped around three hub genes, *EGFR*, *UBQLN1* (ubiquilin1), and *YWHAQ* (14-3-3 protein theta). This cluster was strongly enriched for members of the *EGFR* signaling pathway (Benjamini corrected *P* = 5.57 x 10^−^^5^, supplementary fig. S14, Supplementary Material online), and 20 of the 21 proteins were phosphoproteins (Benjamini corrected *P* = 1.55 x 10^−^^4^). *EGFR* and its downstream kinase cascades play essential roles in general cell growth and differentiation. Their exact relevance in recent coselection is unclear. Other clusters suggested that coselection may impact phosphoprotein-based intracellular signaling cascades (cluster 3) and acetylation-mediated protein transport (cluster 4; supplementary figs. S15 and S16, Supplementary Material online).

Similar overall patterns were observed in CEU. Recent coselection seemed to have substantially affected the nervous system and phosphoprotein-based signaling pathways. In particular, cluster 1 in CEU centered on the growth factor receptor-bound protein (*GRB2*) and the *PTPN11* was highly enriched for phosphorylation, the intracellular signaling cascade and the protein kinase cascade (Benjamini corrected *P* = 6.17 x 10^−^^9^, 9.49 x 10^−^^8^, and 2.56 x 10^−^^5^, respectively, fig. 6*B*). Many of these signaling proteins appeared to contribute to neurotrophin signaling (Benjamini corrected *P* = 2.8 x 10^−^^3^, fig. 6*B*). Neurotrophins are growth factors that promote the growth and survival of neurons. Clusters 2, 3, and 4 in CEU further supported the involvement of the nervous system in recent coselection. In cluster 2, all 17 proteins grouped around *YWHAG* are related to brain tissue (Benjamini corrected *P* = 0.012) and clusters 3 and 4 are both have significant roles in cell junction and neuron-associated functions such as synapse, dendrite, and neuron projection (supplementary figs. S17–S19, Supplementary Material online). Interestingly, we found a subpathway in cluster 4 composed of *CTNNB1*, glycogen synthase kinase 3 beta (*GSK3B*), and microphthalmia-associated transcription factor (*MITF*), which is a part of the melanogenesis pathway (Benjamini corrected *P* = 0.04, supplementary fig. S19, Supplementary Material online). This is a well-known pathway that underwent recent positive selection in CEU (Izagirre et al. 2006). These three genes and the strongest selection signal of Pygopus homolog 1 (*PYGO1*) in cluster 4, were all involved in Wnt signaling pathway (Benjamini corrected *P* = 0.014).

There were few coselection clusters in YRI, likely due to the lower number of candidate genes identified in this population. Cluster 1, centered on *HIF1A*, was enriched in transcription regulatory activity and nucleoside metabolism (Benjamini corrected *P* = 0.002 and 0.02, respectively, fig. 6*C*) and cluster 2 seemed to involve the chemokine signaling pathway and chemokine mediated wounding response, immunity, and defense (supplementary fig. S20, Supplementary Material online).

## Discussion

In this study, we detected the genome-wide evidence of positive selection by integrating the signals from multiple well-established statistics. This is a scheme that is known to enhance the signal specificity and precision of selection center localization (Grossman et al. 2010). The use of genome sequencing data (Abecasis et al. 2012), improved the signal resolution compared with the SNP data used in previous studies (Akey 2009). The dependence of recent genetic adaptations on the functional interactome structure was determined by projecting this list of candidates onto the PPI network.

First, we found a moderate pattern that recent positive selections tended to occur more on the subcentral region of the PPI network, where the DC ranges between 10 and 20 and the BC ranges from 2,000 to 20,000 (fig. 2). This propensity is consistent with a previous study of recent selection in the insulin/TOR signal transduction pathway (Luisi et al. 2012). However, it differs from studies done on a macroevolutionary timescale, which have consistently reported that accelerated evolutionary rates tend to happen at the periphery of the protein interaction network, likely due to the lower functional constraints for the peripheral proteins (Jeong et al. 2001; Fraser et al. 2002). Such inconsistencies may arise by two reasons. First, the dynamics of genetic turnover can differ between macro and microevolution. In microevolution, where population genetic factors are dominant, adaptive selections may happen to more essential genes that have more strength in allowing phenotypic effects in order to efficiently cope with dramatically changing environment in much shorter evolutionary time. These genetic changes are often population specific and their allelic effects can switch between beneficial and deleterious, depending on the diversifying environments. In the long run, when macroevolution plays a major role, many of these local adaptive changes can be transient, either diminishing as the external pressures are lifted, or being replaced by genetic variants that induce less adverse side effects. Second, the higher evolutionary rate in nonessential genes may be due more to relaxation of selection constraints than positive selection. The lack of constraints allows nonessential genes to evolve at a rate close to neutral drift, much higher than that of the functionally important genes, which are constantly under the scrutiny of negative selection. Discrepancies between this and previous studies may also have occurred because the method in this study only considered genes of positive selection, whereas previous studies did not distinguish between evolution acceleration that was due to positive selection and that induced by relaxation.

To date, there have been few systematic studies on relationships between recent selection signals, partially due to the strong stochasticity associated with the genetic polymorphisms at the population genetic scale. Rohlfs et al*.* (2010) proposed to use allelic association between physically unlinked loci to detect ongoing coevolution. However, the test power of this method is low because allelic associations induced by coevolution would instantly vanish when selection ends. In this study, we tested the global dependence of selection signals on the PPI network. We found that positively selected genes tended to lie closer to each other on PPI network than expected under null hypothesis (fig. 3). In particular, there is a highly significant negative correlation (*P* < 10^−^^8^) between the network distance and the chance of that both genes would be positively selected (fig. 3*A*). This negative correlation persists after controlling for network position, short chromosomal distances, and gene length. The obvious pattern that positive selection signals tend to occur in the network vicinity of other selection signals supports the strong impact of gene coselection on recent human history. However, the precise mechanism of coselection is hard to define, while coevolution between two directly interacting proteins or pathway selection where several proteins involved were all affected under the same selective pressure could be two main possibilities that lead to coselection. This study was based on the PPI network, because protein interactome is the most commonly used in studies of coevolution and the physical interactions are the elementary building blocks of any higher order relationships in cellular function (Vidal et al. 2011; Bezginov et al. 2013). Our analyses, based on PPI network, suggest that the selection impacts are likely to co-occur between nearby proteins either via compensatory changes in physical interactions or via protein complex modules in the same pathway under selective pressure. However, other mechanisms of coselection cannot be ruled out, such as gene regulatory networks (Kuo et al. 2010), coexpression networks (Jordan et al. 2004), and metabolic pathways (Chen and Dokholyan 2006). These should be explored in the future studies. More powerful methods featuring selection time and coefficient probably will gain more insights into the mechanisms and potential epistatic interactions of coselection.

Furthermore, recent evolutionary history was examined by the two tests based on divergence trees. In both cases, it was demonstrated that similarity of trifurcate trees decreased as network distance increased (fig. 4). This confirmed that genes closer to each other on the protein–protein interactome tended to influence each other’s divergent evolution toward the same direction. When the confounding factor of LD was taken into consideration, the correlation between RTD and SPL was robust whereas the correlation between Syn score and SPL was not. It should be noted that RTD is based on rank, a nonparametric statistic that is not affected by the divergence score distribution. On the other hand, the Syn score takes the full divergence information into consideration, approaching high values only when two trees are highly similar. It is more difficult to satisfy this condition in the context of microevolution since the differences in selection starting time and selection coefficient both contribute to the differentiation of divergence trees between coselected genes.

Whether coevolution or pathway selection dominated the cases of coselection, it is difficult to answer. Ralph and Coop (2010) studied parallel adaptation with simulations in a continuous, geographically spread population. They assume that there may be many waves of selection acting on multiple advantageous alleles of similar phenotypic effect in parallel. They showed that under some specific conditions such polygenic model would give rise to parallel selection on the course of functional adaptation. Analyses of genome-wide association studies (GWASs) may also shed light to this discussion. GWAS studies usually identify multiple loci that individually explain a fraction of the total phenotypic variance. If there are strong epistatic interactions among the loci, then the total phenotypic variance accounted for by all loci together would be greater than simple linear sum of individual loci’s effects, otherwise the two terms should equal. Various additive and epistatic models have been proposed to account for the architecture of complex traits and the phenomenon of missing heritability (Gibson 2011). Some traits, such as body height seem to fit into models assuming noninteractions (Yang et al. 2010), and focus on the potential of rare variants to explain additional variances. However, more and more attentions have been paid to nonadditive epistasis, gene-environment interactions, and epigenetic effects (Mackay et al. 2009; Manolio et al. 2009). Recent coselections of complex traits may have affected the underlying pathways in similar additive/epistatic modes depending on the traits under selection. A recent study showed that under selection, the nonadditive genetic variants may persist longer evolutionary period than the additive genetic variations, suggesting that epistatic effects could have played a major role in quantitative traits (Hemani et al. 2013). To be able to clearly distinguish between coevolution and parallel selection in the same pathway, more powerful tests that detect the potential epistatic interactions between coselection signals are needed.

It is advantageous to be able to identify genes that were coselected during recent human history. However, the methods used in this study were not powerful enough to test the coselection between specific pairs of genes. We therefore partially addressed this problem by identifying clusters of genes that showed signs of strong evolutionary correlations, for example, high Syn scores, and located only one-step away from each other. Interestingly, these clusters demonstrated strong functional specificities according to the enrichment analysis. In particular, many significant categories are related to the nervous system, including synapse, neurotrophin, and brain, and the proteins involved often constitute different parts of the signaling cascades. Other enriched categories that were found, such as B-cell activation and inflammatory response, were related to the immune system (fig. 6 and supplementary figs. S14 and S20, Supplementary Material online). The finding of recently coselection subnetworks that involved the nervous and immune systems was not unexpected because these two systems have also been reported as being enriched with signals of recent positive selection in different human populations (Pickrell et al. 2009; Grossman et al. 2013). However, many well-known signals of positive selection did not appear in the candidate clusters of coselection. For instance, *SLC24A5* is associated with skin pigmentation, *LCT* is associated with dairy farming in CEU (Bersaglieri et al. 2004; Lamason et al. 2005), and *EDAR* affects the hair and sweat glands in EAS (Kamberov et al. 2013). There are several possibilities for the fact that these signals were not present. First, the adaptive genetic changes in these genes likely did not induce compensatory changes in their network neighbors or these individual genes have such large effects on adaptive phenotypes that pathway selection would not be necessary to happen or their effects of being coselected were too small to be detected by our method. It should be noted that our current method tended to identify coselection subnetworks that consisted of relatively large numbers of proteins that closely interacted with each other within one step. This would bias the coselection signals toward subnetworks with higher connectivity, such as the signaling pathways reported in our results. Second, the coselection of these genes may have occurred at other interaction levels, such as metabolic networks or gene regulation so that the coselected partners did not necessarily appear as interacting neighbors on the PPI network. *LCT* seems to be such case since its main role is in the galactose metabolism pathway, which is not reflected in the PPI network. In such cases, recent coselection in various types of biological networks should be explored. Third, authentic PPIs in vivo depend on specific biological contexts (e.g., tissue specificity, environmental conditions) and involve a wide spectrum of mechanisms. However, the current PPI database was mainly constructed from high-throughput Y2H screens followed by manual curation, which did fully account for diverse biological conditions (Bruckner et al. 2009; Szklarczyk et al. 2011). Thus, the current PPI database is not complete and therefore it is inaccurate to a certain degree. This may explain the cases of *SLC24A5* and *EDAR*. *SLC24A5* is a membrane transporter that plays an essential role in melanogenesis (Ginger et al. 2008). However, the PPI database failed to identify any partners that interacted with it. A similar situation was found with *EDAR*, a membrane receptor protein, which also had no partner interactions on the PPI network. In fact, *EDAR* directly interacts with its ligand, *EDA* and its adaptor, *EDARADD* in *NF-κB* signaling pathway and contributes to many aspects of ectodermal development, including development of hair, teeth, and sweat glands (Mikkola and Thesleff 2003). This suggests that the future fine characterization of the coselection subnetworks should be based on more comprehensive and accurate network databases.

Overall, this study has provided insight into recent coselection, since humans migrated from Africa. Our findings have provided the first genome-wide evidence that coselection has strongly shaped recent human history and have indicated that many signals of recent selection may have been coselected either due to compensatory coevolution or parallel pathway selection. However, these findings are preliminary. Future studies that enhance the resolution of the selection signals or that develop more powerful methods are needed to better identify the coselected genes and to infer the mechanisms of recent coselection.

## Supplementary Material

Supplementary tables S1–S3 and figures S1–20 are available at *Genome Biology and Evolution* online (http://www.gbe.oxfordjournals.org/).

## Funding

This work was supported by the Cutting Edge Research Project from the CAS-SIBS Outstanding Junior Researchers [2011KIP201]; the Key Research Direction from the CAS Knowledge Innovation Project [KSCX2-EW-Q-1-12]; the Max-Planck-Gesellschaft Partner Group Grant; and National Natural Science Foundation of China [31371267]. The funders had no role in study design, data collection or analysis, decision to publish, or preparation of the manuscript.

## Supplementary Material

Supplementary Data
