# Supplementary material for: Recent Coselection in Human Populations Revealed by Protein–Protein Interaction Network
Source: Genome Biol Evol. 2014 Dec 21;7(1):136–53. doi: 10.1093/gbe/evu270 (PMC4316623; doi:10.1093/gbe/evu270)
Supplement: Supplementary Data [file supp_7_1_136__index.html]

Recent Coselection in Human Populations Revealed by Protein–Protein Interaction Network — Supplementary Data 

# Recent Coselection in Human Populations Revealed by Protein–Protein Interaction Network

## Supplementary Data

files

**Files in this Data Supplement:**

- Supplementary Data - docx file
- Supplementary Data - rar file
