# Supplementary material for: Recent Coselection in Human Populations Revealed by Protein–Protein Interaction Network
Source: Genome Biol Evol. 2014 Dec 21;7(1):136–53. doi: 10.1093/gbe/evu270 (PMC4316623; doi:10.1093/gbe/evu270)
Supplement: Supplementary Data [file supp_evu270_New_Microsoft_Office_Word_Document.docx]

**Supplementary Materials**

Supplementary materials, Table S1-S3, Figure S1- S20 are available at Genome Biology and Evolution online.

**Table S1. A list of candidate genes under recent positive selection in EAS, CEU and YRI identified by the CMS method. (XLS)**

**Table S2. Statistical p values based on permutation analyses and multiple testing correction. (XLS)**

**Table S3. Correction for confounding factors of network position, genomic position and gene length. (XLS)**

**Figure S1-S4. Average SPL of co-selection, compared with those under null distribution of 10,000 random samplings, in YRI, CEU, EAS and the three populations combined. (PDF)**

**Figure S5-S9. Relative proportion score of co-selection and enrichment Z-score over SPL after correcting for network position, genomic position and gene length. (PDF)**

**Figure S10-S11. Relationship between the similarity of divergence trees and network distances of SPL after correcting for physically genomic position. (PDF)**

**Figure S12-S13. Sub-networks revealed co-selection patterns in Europeans and Yorubans. (PDF)**

**Figure S14-S20. Functional enrichment analysis of co-selection clusters in EAS, CEU and YRI. (PDF)**
